# A survey of evidence users about the information need of acupuncture clinical evidence

**DOI:** 10.1186/s12906-016-1434-0

**Published:** 2016-11-10

**Authors:** Xiue Shi, Xiaoqin Wang, Yali Liu, Xiuxia Li, Dang Wei, Xu Zhao, Jing Gu, Kehu Yang

**Affiliations:** 1 Evidence-Based Medicine Center, School of Basic Medical Sciences, Lanzhou University, Lanzhou, 730000 China; 2 Key Laboratory of Evidence Based Medicine and Knowledge Translation of Gansu Province, Lanzhou, 730000 China; 3 Chinese GRADE Center, Lanzhou, 730000 China; 4 Gansu Rehabilitation Center Hospital, Lanzhou, 730000 China; 5 Department of Hypertension, Lanzhou University Second Hospital, Lanzhou, 730000 China; 6 Gansu University of Chinese Medicine, Lanzhou, 730000 China

**Keywords:** Acupuncture SR/MAs, Information need, Questionnaire, Evidence users

## Abstract

**Background:**

The PRISMA statement was rarely used in the field of acupuncture, possibly because of knowledge gaps and the lack of items tailored for characteristics of acupuncture. And with an increasing number of systematic reviews in acupuncture, it is necessary to develop an extension of PRISMA for acupuncture. And this study was the first step of our project, of which the aim was to investigate the need for information of clinical evidence on acupuncture from the perspectives of evidence users.

**Methods:**

We designed a questionnaire based on a pilot survey and a literature review of acupuncture systematic review or meta-analysis(SR/MA). Participants from five cities (Lanzhou, Chengdu, Shanghai, Nanjing and Beijing) representing the different regions of China, including clinicians, researchers and postgraduates in their second year of Master studies or higher level, were surveyed.

**Results:**

A total of 269 questionnaires were collected in 18 hospitals, medical universities and research agencies, and 251 (93 %) with complete data were used for analysis. The average age of respondents was 33 years (SD 8.959, range 25–58) with male 43 % and female 57 %. Most respondents had less than 5 years of working experience on acupuncture, and read only one to five articles per month. Electronic databases, search engines and academic conferences were the most common sources for obtaining information. Fifty-six percent of the respondents expressed low satisfaction of the completeness of information from the literature. The eight items proposed for acupuncture SR/MAs received all high scores, and five of the items scored higher than eight on a scale zero to ten. The differences for the scores of most items between postgraduates and non-postgraduates were not statistically significant.

**Conclusions:**

The majority of the respondents were not very satisfied with the information provided in acupuncture SRs. Most of the items proposed in this questionnaire received high scores, and opinions from postgraduates and non-postgraduates tended to agree on most items. Comments from the respondents can promote future work.

**Electronic supplementary material:**

The online version of this article (doi:10.1186/s12906-016-1434-0) contains supplementary material, which is available to authorized users.

## Background

Reporting guidelines promote transparent and rigorous reporting. In 1996, several experts from the United States, United Kingdom and Canada worked together and published the Consolidated Standards of Reporting Trials (CONSORT) statement in Journal of the American Medical Association (JAMA), which started the rapid development of reporting guidelines in medical research [1]. With the evolving methodology of randomized controlled trials (RCT) and evidence-based medicine (EBM), CONSORT has been updated in 2001 and 2010 [2, 3], CONSORT 2010 being the latest version. CONSORT has been endorsed and uptaken after its release by several medical journals [4], and reporting guidelines have improved the quality of both the reporting and the methodology [5, 6]. Because different fields of medicine and different types of research data differ in characteristics, several extensions have been developed. The *STandards for Reporting Interventions in Clinical Trials of Acupuncture* (STRICTA) was officially published in 2001, and was updated along with CONSORT in 2010 [7, 8]. Authors of acupuncture trials and systematic reviews have expressed their belief that STRICTA substantially contributes to the reporting of acupuncture interventions [9] and has significantly improved the reporting quality [10].

High-quality systematic reviews (SRs) of acupuncture form the best evidence to inform guidelines and clinical practice. Accurate and complete reporting enables readers to determine the internal and external validity of the research result, and editors and reviewers to make comprehensive and objective judgement effectively. The Preferred Reporting Items for Systematic reviews and Meta-Analyses (PRISMA) statement was developed for systematic reviews and meta-analyses (SR/MAs) [11]. Although PRISMA promotes the quality of the reporting and methodology of SR/MAs, in some specific areas, it cannot meet all the needs. The extensions PRISMA-Equity [12], PRISMA-Abstract [13], PRISMA-Protocol [14] and PRISMA-Network Meta-Analysis [15] were developed in 2012, 2013, 2015 and 2015, respectively. In 2012, the assessment of the published 476 acupuncture SRs/MAs with PRISMA and their included RCTs showed low quality in general. Information from those studies could not meet the needs of acupuncture practitioners, and therefore was not usable for implementation [16]. Furthermore, the results from a survey of the application status of the PRISMA statement indicated that it was rarely used in the field of acupuncture, possibly because of knowledge gaps among researchers. Another probable reason was lack of items tailored for the characteristics of acupuncture [17]. On the other hand, research in acupuncture develops rapidly with an increasing number of studies published every year [18]. Thus, it is necessary to develop an extension of PRISMA for acupuncture, and implement it along with PRISMA.

Our project aims to develop a reporting guideline for acupuncture SR/MAs. This project consists of three phases: the investigation about the requirements of evidence users for the information needs of reporting of acupuncture SR/MAs; three rounds of Delphi process; and a face-to-face meeting for reaching consensus. This paper described the first phrase, where we aimed to conduct a survey among the evidence users about their information needs, to directly capture their opinions about the current reporting of evidence and the further needs on information reported in acupuncture SR/MAs. Before this survey, we conducted a pilot study in Lanzhou on the questionnaire, and found that most of the questions and items were highly valued by the participants. We also investigated the reporting rates of the items in acupuncture SR/MAs, and found a range of 6.3 to 73.7 % [19]. In this survey, with comments from users and evidence from the literature review, we aimed to design a rigorous and valid questionnaire, and to conduct a survey among acupuncture clinicians, researchers, teachers, and postgraduates across the whole country. Based on this survey, we will draft the items for acupuncture SR/MAs reporting for the Delphi process in next period.

## Methods

### Design of the questionnaire

We designed a questionnaire containing three main parts: 1) demographic information of the respondent, including sex, age, occupation, education, and professional title; 2) the experience, awareness and knowledge on the clinical evidence of acupuncture, including the duration of career, working fields, and reading behavior, 3) the importance of the proposed items from the perspectives of evidence users, including background information about acupuncture, diagnostic criteria of Chinese medicine, types and details of acupuncture, outcome measures of the effects, experience of the operators, and the rationale of the follow-up time. Furthermore, respondents were invited to provide any item they thought important but were missing in our list. The initial items in part three were based on the analysis of existing reporting guidelines, such as STRICTA and PRISMA, and a systematic review of 476 SR/MAs on acupuncture [16] (see Additional file 1 for a copy of the questionnaire).

The questions were designed to be easy to complete, with several pre-defined options from which the respondents could simply choose their answer. An open-ended response marked “other” was added into some questions if necessary. Respondents were asked to score the reporting items for the necessity and feasibility of inclusion in SR/MAs of acupuncture using the Likert scale [20] ranging from zero (not necessary) to ten (essential). For the eight items need to be rated, each question included a space for “explanation” in the end for respondents who were willing to provide more details.

### Method to conduct survey

We conducted a pilot survey by two trained investigators (XQW, DW) in Lanzhou to find out necessary adjustment and to evaluate the feasibility in an attempt to predict an appropriate sample size and improve the study design prior to performing the formal one. Then, the two trained investigators completed the formal survey between April and June, 2014 by distributing and collecting the questionnaires in person, which could prevent the low response rate of electronic questionnaire. Our investigators were responsible only for distributing the material, interpreting the background, and checking with the respondents to ensure the completeness of the questionnaires. For subjective questions, the respondents were asked to fill it themselves without our investigators’ interfering.

In the formal survey, we visited Traditional Chinese Medicine agencies, hospitals and colleges in five cities (Lanzhou, Chengdu, Shanghai, Nanjing and Beijing). With the inclusion criterion that respondents must have acupuncture clinical experience more than 1 year, we surveyed clinicians, researchers, postgraduates in their second year or higher, and doctors majoring in acupuncture. We surveyed at least 50 individuals in each city, with about 40 % of respondents being students. Acupuncture students, practitioners and researchers have a critical role in bridging evidence and practice, and their opinions are mainly based on practical work which can provide valuable experience on what is the most urgently needed information in acupuncture SR/MAs. Before we distributed the questionnaire, we identified a liaison person in every city, and collected information about the respondents in advance. Then, the investigator visited the respondents one by one. This investigation was approved by the Research Ethics Committee of the First Hospital of Lanzhou University, Lanzhou, China (approval number: LDYYL2013-0007). All the participants were required to sign informed consent. We marked student or non-student on the left top of each questionnaire to avoid errors.

### Data analysis

Data were processed using Epidata 3.1 and analyzed with software Excel 2013. For the close-ended question with given options, we used frequencies and percentages to summarize the results. For evaluating the importance of the proposed items, we used mean value of the score, and analyzed the difference between postgraduates and non-postgraduates (including clinicians and researchers) with independent-Sample *T*-test with the SPSS Statistics 19 software. Additional items provided by respondents beyond this questionnaire were listed in a table. We abstracted all the questionnaire independently and grouped them according to the type of suggestion or opinion on reporting. All discrepancies were discussed and agreed on for final interpretation.

## Results

**Fig. 1:**
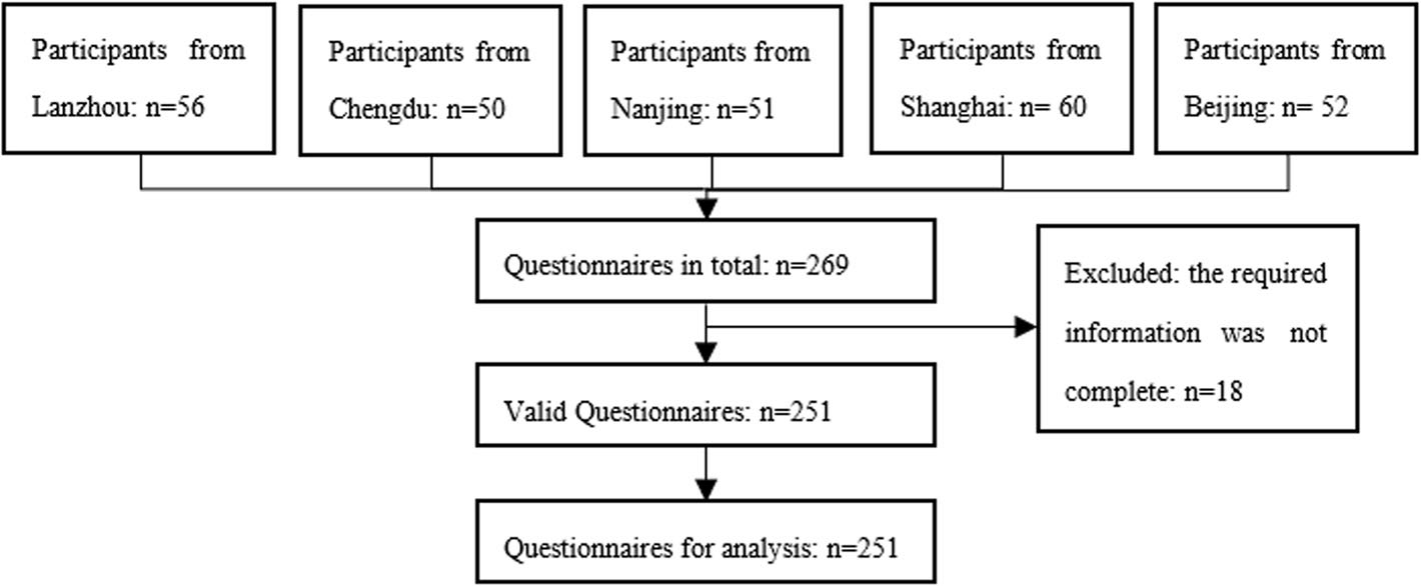
Flow chart of the survey, including the source of the respondents and the process of identifying valid questionnaires

A total of 269 questionnaires were collected, of which 251 (93 %) were used for analysis and 18 (7 %) were excluded because of missing data (Fig. 1). Of the included questionnaires, 52 (21 %) were from Lanzhou, 50 (20 %) from Chengdu, 48 (19 %) from Nanjing, 55 (22 %) from Shanghai, and 46 (18 %) from Beijing. The demographic and socio-economic characteristics of the respondents are shown in Table 1. The average age was 33 years (SD = 9.0, range 25–58).

**Table 1:** Characteristics of the respondents

Variable	Categories	Respondents: *n* (%)
Total		251
Age	20 ~ 30	130 (52 %)
	30 ~ 40	75 (30 %)
	40 ~ 50	32 (13 %)
	≥50	14 (6 %)
Sex	Male	142 (57 %)
	Female	109 (43 %)
Education	Medical Doctor	42 (17 %)
	Master	161 (64 %)
	Bachelor	46 (18 %)
	Beneath Bachelor	2 (1 %)
Occupation	Postgraduate student specializing in acupuncture	95 (38 %)
	Clinician practicing acupuncture	126 (50 %)
	Others (including teachers and researchers)	30 (12 %)
Health worker grade^a^	Senior	39 (16 %)
	Vice-senior	30 (12 %)
	Middle	43 (17 %)
	Primary or below	51 (20 %)
	No title	88 (35 %)
Specialty	Western Medicine	3 (1 %)
	Traditional Chinese Medicine (TCM)	236 (94 %)
	Integrative Medicine	12 (5 %)

^a^This refers to the category of title obtained after passing a qualifying test

Table 2 showed the findings on the respondents’ experience and knowledge on clinical evidence of acupuncture. More than half of the respondents had work experience of less than 5 years. The main work of most respondents was clinical treatment with acupuncture. On the other hand, a notable percentage of the participants were involved in more than three types of work. Most of the respondents read one to five articles each month. According to the response, electronic databases, printed professional journal, academic conferences, search engines, and ancient literature were all commonly used to obtain information. Forty-three of the respondents chose at least three or more approaches. The most popular type of literature read by the respondents was RCT, and for the satisfaction of the completeness of information in acupuncture literature, only 2 % were very satisfied.

**Table 2:** Findings on respondents’ experience and knowledge on clinical evidence of acupuncture

Subjects	Options	Respondent: *n* (%)		
		Postgraduates (95)	Non-postgraduates^b^ (156)	Total
Years of experience in acupuncture	<5 years	79 (83 %)	49 (31 %)	128 (51 %)
	5-10 years	14 (15 %)	46 (29 %)	60 (24 %)
	10-20 years	1 (1 %)	25 (16 %)	26 (10 %)
	>20 years	1 (1 %)	36 (24 %)	37 (15 %)
Main work related to acupuncture (multiple answers allowed)	Clinical treatment	87 (92 %)	141 (90 %)	228 (91 %)
	Rehabilitation care	26 (27 %)	45 (29 %)	71 (28 %)
	Clinical research	49 (52 %)	85 (54 %)	134 (53 %)
	Basic research	17 (18 %)	37 (24 %)	54 (22 %)
	Review	12 (13 %)	16 (10 %)	28 (11 %)
	Writing SR/MA	9 (9 %)	10 (6 %)	19 (8 %)
	Writing clinical guidelines	2 (2 %)	6 (4 %)	8 (3 %)
	Acting as a reviewer	0 (0 %)	8 (5 %)	8 (3 %)
	Editor	0 (0 %)	1 (0.4 %)	1 (0.4 %)
	Others	0 (0 %)	3 (2 %)	3 (1 %)
Papers on acupuncture read per month	>10	17 (18 %)	35 (22 %)	52 (21 %)
	5–10	25 (26 %)	42 (27 %)	67 (27 %)
	1–5	48 (51 %)	76 (49 %)	124 (49 %)
	0	5 (5 %)	3 (2 %)	8 (3 %)
Ways of obtaining information on acupuncture (multiple answers allowed)	Medical databases	83 (87 %)	130 (83 %)	213 (85 %)
	Printed professional journals	21 (22 %)	65 (42 %)	86 (34 %)
	Academic meetings	30 (32 %)	82 (53 %)	112 (45 %)
	Search engines	40 (42 %)	79 (51 %)	119 (47 %)
	Ancient literature bibliographies	38 (40 %)	61 (40 %)	99 (39 %)
	Others	1 (1 %)	3 (2 %)	4 (16 %)
Types of studies most commonly read (multiple answers allowed)	RCTs	68 (72 %)	104 (67 %)	172 (69 %)
	Observational studies	52 (55 %)	75 (48 %)	127 (51 %)
	Basic research	37 (39 %)	78 (50 %)	115 (46 %)
	Reviews	32 (34 %)	48 (31 %)	80 (32 %)
	SRs/MAs	29 (31 %)	63 (0 %)	92 (37 %)
	Clinical practice guidelines	25 (26 %)	65 (42 %)	90 (36 %)
	Ancient literature	27 (28 %)	61 (39 %)	88 (35 %)
	Others	1 (1 %)	3 (2 %)	4 (2 %)
Satisfaction of the information acquired from the literature	Very satisfied	1 (1 %)	1 (1 %)	2 (2 %)
	Satisfied	56 (59 %)	85 (54 %)	141 (56 %)
	Sometimes satisfied	28 (29 %)	54 (35 %)	82 (33 %)
	Not satisfied	10 (11 %)	16 (10 %)	26 (10 %)

^b^Non-postgraduates include clinicians and researchers

For the reporting items in our questionnaire, mean scores were all above five. Table 3 presents the respondents’ ranking of each item. The additional suggestions proposed by the respondents about what should be presented in acupuncture SR/MAs were shown in Table 4.

**Table 3:** Scores of the candidate items

Score		1	2	3	4	5	6	7	8	9	10	Mean	*p *(independent-sample *T*-test)
7.1	Total	6	9	14	17	24	18	18	48	25	72	7.2	
	P^c^	2	5	1	5	9	7	7	15	11	33	7.6	0.109
	NP^d^	4	4	13	12	15	11	11	33	14	39	7.0	
7.2	Total	19	11	14	15	33	18	23	37	21	60	6.6	
	P	9	3	4	5	12	8	8	13	13	20	6.6	0.895
	NP	10	8	10	10	21	10	15	24	8	40	6.6	
7.3	Total	3	2	0	4	18	15	9	40	33	127	8.6	
	P	1	1	0	0	7	6	5	16	13	46	8.5	0.954
	NP	2	1	0	4	11	9	4	24	20	81	8.6	
7.4	Total	2	0	2	4	15	9	12	46	45	116	8.6	
	P	1	0	1	1	9	2	1	19	18	43	8.6	0.700
	NP	1	0	1	3	6	7	11	27	27	73	8.7	
7.5	Total	1	0	3	9	12	8	14	37	42	125	8.7	
	P	1	0	2	2	7	4	8	15	15	41	8.4	0.044
	NP	0	0	1	7	5	4	6	22	27	84	8.8	
7.6	Total	1	2	3	6	8	13	16	42	42	118	8.6	
	P	1	2	0	2	2	6	5	19	16	42	8.5	0.560
	NP	0	0	3	4	6	7	11	23	26	76	8.7	
7.7	Total	17	11	25	17	38	36	20	45	16	26	5.9	
	P	11	3	9	7	18	12	7	17	4	7	5.5	0.047
	NP	6	8	16	10	20	24	13	28	12	19	6.2	
7.8	Total	3	2	5	8	14	24	21	55	34	85	8.0	
	P	2	1	2	3	5	13	6	20	13	30	7.8	0.286
	NP	1	1	3	5	9	11	15	35	21	55	8.1	

^c^P: Postgraduates; ^d^NP: Non-postgraduates, including clinicians and researchers of acupuncture

7.1 Provide the theoretical basis of acupuncture used for the target disease in background/introduction

7.2 Provide the style of acupuncture treatment (e.g. traditional Chinese acupuncture, South Korean acupuncture) in background/introduction

7.3 Provide the diagnostic criteria in methods (TCM syndrome and/or diagnostic criteria of diseases according to Western medicine)

7.4 Provide types of acupuncture interventions in methods (e.g. type of acupunctures like percussopunctator and needles, and any other intervention like sham acupuncture)

7.5 Provide details of acupuncture interventions in methods (e.g. number of needles, names of acupoint, depth of puncture, relevant body response, needling manipulation, time for needle retention, types of needles)

7.6 Provide indicators of effect judgement in methods (e.g. Visual Analogue Scale (VAS))

7.7 Provide the qualification (e.g. career and other experience) of acupuncture clinicians in methods

7.8 Provide the follow-up time along with rationality in results

**Table 4:** Additional items proposed by respondents

Items	Content	Respondents (n)
7.9.1	Specify whether the included studies were RCTs and describe the method of randomization.	10
7.9.2	Specify the statistical and data processing methods, and estimate the statistical power of the data analysis.	8
7.9.3	Discuss the rationale and mechanism of action for acupuncture in modern medicine.	7
7.9.4	Describe the safety and adverse effects (both long term and short term) of acupuncture, such as epilepsy.	7
7.9.5	Provide the effect and reproducibility of acupuncture, including the description of effect evaluation time and indicators and patient outcomes.	7
7.9.6	Describe the demographic information (e.g. age, sex), physical status, medical history and conditions.	5
7.9.7	Describe the method, including study design, quality assessment, and bias control.	4
7.9.8	Describe whether the preferences and values of the patients were considered or not.	3
7.9.9	Describe the follow-up time, result, and the loss to follow-up and corresponding solutions.	3
7.9.10	Describe the origin and development of acupuncture.	3
7.9.11	Describe the status quo of research on diseases and acupuncture both at home and abroad.	3
7.9.12	Analyzing the results and presenting the discussion.	3
7.9.13	Description of the blinding method of the included studies.	2
7.9.14	Providing the references of the study.	2
7.9.15	Description of the limitations and implications of the study.	2
7.9.16	Description of the sample method and size.	1
7.9.17	Description of the innovations and strengths of the study.	1
7.9.18	Description of which type of clinical research is suitable for acupuncture.	1
7.9.19	Description of the Ethics Committee review and registration status.	1
7.9.20	Description of the conclusion of the study and the clinical evidence on acupuncture.	1
7.9.21	Declaration of the funding of the study.	1
7.9.22	Description of the environment of study implementation (such as primary health setting or one of the top three hospitals).	1
7.9.23	Description of the standard of syndrome differentiation and treatment.	1

In addition to the eight items proposed in our questionnaire, some respondents also gave suggestions on other information

## Discussion

Our survey and questions were designed for acupuncture evidence users with the hope of collecting reporting items for acupuncture SR/MAs, which was different from STRICA for reporting interventions in clinical trials of acupuncture. To ensure the representativeness, we discussed how to select the participants considering various aspects, including geographic location, gender, and work experience. We visited participants from five cities located in south, north, north-west and south-west China, and included about 40 % students. We surveyed students who were at least in the second year of their Master studies, and having completed an internship on acupuncture for at least one year, to avoid surveying participants whose knowledge of acupuncture is too limited to provide practical response. We recruited the participants from universities conducting research and teaching in TCM and their affiliated hospitals, research institutes for acupuncture, and TCM hospitals, to be able to fully reflect the need of scientific research among the various groups of evidence users.

Our findings were not optimistic in terms of the reading behavior of the respondents. Most of the respondents read one to five relevant articles per month, and surprisingly, we found also respondents who never read the literature. Yet most of the participants could give advice about the information they need in an article, based on their clinical experience of acupuncture. Forty-three percent of the respondents were not satisfied with the adequacy of information reported in SR/MAs, which corresponds to the poor reporting quality of acupuncture SR/MAs [16].

Acupuncture is used for various kinds of diseases [21] which differ from each other in terms of theoretical and evidence basis. The authors should clearly describe the theoretical knowledge on the use of acupuncture for this disease to promote the rapid understanding for readers. Acupuncture is characterized by a broad diversity of styles and approaches in both East Asia and Western countries [22], and the ways of conducting acupuncture and the theories behind these different styles also differ [23]. Therefore the appropriate background information on the style, and theoretical basis of acupuncture for the specific disease should be reported.

In general, there are two different sets of diagnostic criteria: syndrome differentiation used in Traditional Chinese Medicine, and diagnostic criteria of diseases used in Western medicine [24]. These criteria should be interpreted in completely different ways, and it is important to indicate which set of criteria, or both, should be used. There are diverse types of needles, and nine classical acupuncture needles are always mentioned, which include filiform needle, shear needle, round-pointed needle, spoon needle, lance needle, round-sharp needle, stiletto needle, long needle and big needle [25]. With different functions [26], it is necessary to state which type of needles the study can be applied to. At the same time, the details of the acupuncture intervention including numbers of needles, names of acupoints, depths of puncture, de-qi or not, and times for needle retention, will all influence the treatment effect. As for judgement on effect, there are many indicators used only in acupuncture, such as acupuncture-tailored symptomatic relief and adverse effects like bleeding. Between 2000 and 2009, 95 cases of serious adverse events were reported [27]. However, many such events have not been reported among adequately-trained acupuncturists, and they should therefore not be considered inherent to acupuncture, but instead being due to malpractice of acupuncturists [28]. Therefore, a qualified acupuncturist is needed to ensure the effectiveness and safety of the treatment. The necessary follow-up time differs greatly between different conditions, and the patients treated with acupuncture also need to be followed up for possible side effects.

Our results showed a high acceptability and recognition for most items among the respondents (Table 3). The mean scores of the third, fourth, fifth, sixth and eighth item were higher than eight, the first item was 7.2, while items two and seven were lower than six. But we did not exclude or include any items in this period. Instead, we would like to integrate these items with additional comments from the respondents in order to form the checklist of items for the following Delphi process. We also compared the mean scores between postgraduates and NPs (including methodologists, acupuncture clinicians, researchers and teachers), using the independent-sample *T*-test. The results of the *T*-test showed that the difference of the mean scores between postgraduates and NPs were non-significant (*p* value ≥0.05) for six of the eight items, and the *p-*value was close to 0.05 also for the two remaining ones where the difference was found significant (7.5 and 7.7). This indicated that the experienced acupuncture workers and the less experienced postgraduates tend to put similar attention on the most items, that is, both groups demanded more complete information in acupuncture SR/MAs. For details of acupuncture interventions and qualification of acupuncture clinicians in methods, postgraduates gave higher scores compared with non-postgraduates, which might be related with their experience.

For additional comments in Table 4, the top five mentioned items (mentioned by seven or more respondents) were carefully analyzed. The 7.9.1 and 7.9.2 were not tailored for acupuncture, and they have already been mentioned in PRISMA. As for the 7.9.3 and 7.9.5, they were similar or close to 7.1 and 7.4 respectively. And 7.9.4 will be integrated in the item checklist in the future period.

Our study has both strengths and limitations. We designed the questionnaire based on a pilot study and a literature review, combining the two key factors in evidence based medicine—— evidence and practitioners’ experience [29]. We designated two researchers to distribute and collect printed questionnaires and some explanatory materials without interfering the answer process, which helped us improve the response rate [30, 31]. We proposed eight items collected from current literature in our questionnaire, which probably does not cover all relevant issues for reporting SR/MAs of acupuncture. However, the open ended comments from the respondents gave us useful suggestions for further improvement. We limited our survey to five cities, but these cities represent in the different regions of China. We also considered the representative from the aspects of gender, geography, clinical experience, and experience in literature. The participants are representative of typical users of systematic reviews on acupuncture. Acupuncture is mainly practiced in specialized and general hospitals, where the health care workers are in general well educated and involved in research. In China, acupuncture is not commonly practiced in local health centers or private clinics, where the health care workers tend to be less well educated [32, 33]*.* In the next period, relevant experts will be identified and surveyed with an online questionnaire, and the results of this survey, including the comments and additional items suggested by the respondents, will be properly considered and integrated.

## Conclusions

Most clinicians, researchers and students involved in acupuncture were not satisfied with the information provided in acupuncture SRs. This indicates the need for promoting the more complete and critical information reporting of acupuncture SR/MAs. Most of the items proposed in the questionnaire scored highly, and there were only small, mostly non-significant, differences between the opinions from postgraduates and more experienced NPs. The open-ended questions for respondents for collecting comments and additional items provided us with a lot information, which would promote our work in next step.

## Additional file

Additional file 1: The copy of our questionnaire. (DOC 44 kb)

## Abbreviations

CONSORT: Consolidated Standards of Reporting Trials
EBM: Evidence-based medicine
JAMA: Journal of the American Medical Association
PRISMA: Preferred Reporting Items for Systematic reviews and Meta-Analyses
RCT: Randomized controlled trial
SR/MA: Systematic review or meta-analysis
STRICTA: STandards for Reporting Interventions in Clinical Trials of Acupuncture
TCM: Traditional Chinese medicine

## References

[1] Centre for Reviews and Dissemination (2009). Systematic reviews: CRD’s guidance for undertaking reviews in health care.

[2] Moher D, Schulz KF, Altman DG (2001). The CONSORT statement: revised recommendations for improving the quality of reports of parallel-group randomised trials. Lancet.

[3] Schulz KF, Altman DG, Moher D (2010). CONSORT 2010 statement: updated guidelines for reporting parallel group randomised trials. BMC Med.

[4] Endorsers. http://www.consort-statement.org/about-consort/endorsers. Accessed 21 Jan 2015.

[5] Mannocci A, Saulle R, Colamesta V, D'Aguanno S, Giraldi G, Maffongelli E, et al. What is the impact of reporting guidelines on Public Health journals in Europe? The case of STROBE, CONSORT and PRISMA. J Public Health (Oxf). 2014;37(4):737–40. 10.1093/pubmed/fdu108 25538144

[6] Panic N, Leoncini E, de Belvis G, Ricciardi W, Boccia S (2013). Evaluation of the endorsement of the preferred reporting items for systematic reviews and meta-analysis (PRISMA) statement on the quality of published systematic review and meta-analyses. PLoS ONE.

[7] Wu TX, representative (2010). Revised STandards for Reporting Interventions in Clinical Trials of Acupuncture (STRICTA): extending the CONSORT statement. Chin J Evid-based Med.

[8] Extensions. http://www.consort-statement.org/extensions? ContentWidgetId = 559. Accessed 21 Jan 2015.

[9] Prady SL, MacPHERSON H (2007). Assessing the utility of the standards for reporting trials of acupuncture (STRICTA): a survey of authors. J Altern Complement Med.

[10] Hammerschlag R, Milley R, Colbert A, Weih J, Yohalem-Ilsley B, Mist S, Aickin M. Randomized controlled trials of acupuncture (1997–2007): an assessment of reporting quality with a CONSORT-and STRICTA-based instrument. Evid Based Complement Alternat Med. 2011;2011:25. Article ID 183910. doi:10.1155/2011/183910. 10.1155/2011/183910 PMC2952291 20953418

[11] Moher D, Liberati A, Tetzlaff J, Altman DG, PRISMA Group (2009). Preferred reporting items for systematic reviews and meta-analyses: the PRISMA statement. Ann Intern Med.

[12] Welch V, Petticrew M, Tugwell P, Moher D, O'Neill J, Waters E (2012). PRISMA-Equity 2012 extension: reporting guidelines for systematic reviews with a focus on health equity. PLoS Med.

[13] Beller EM, Glasziou PP, Altman DG, Hopewell S, Bastian H, Chalmers I (2013). PRISMA for Abstracts: reporting systematic reviews in journal and conference abstracts. PLoS Med.

[14] Moher D, Shamseer L, Clarke M, Ghersi D, Liberati A, Petticrew M (2015). Preferred reporting items for systematic review and meta-analysis protocols (PRISMA-P) 2015 statement. Syst Rev.

[15] Hutton B, Salanti G, Caldwell DM, Chaimani A, Schmid CH, Cameron C (2015). The PRISMA extension statement for reporting of systematic reviews incorporating network meta-analyses of health care interventions: checklist and explanations. Ann Intern Med.

[16] Liu Y, Zhang R, Huang J, Zhao X, Liu D, Sun W (2014). Reporting Quality of Systematic Reviews/Meta-Analyses of Acupuncture. PLoS ONE.

[17] Wang XQ, Wei D, Liu YL, Wu CL, Ji K, Wei JQ (2014). A survey of application status of the PRISMA statement. Chin J Evid-based Med.

[18] Wang C, He WJ, Guo Y (2010). Analysis on acupuncture related articles published in periodicals in Science Citation Index(SCI)in 2008. Zhongguo Zhen Jiu.

[19] Liu YL. Research on the Quality of Systematic Reviews and Randomized Controlled Trials of Acupuncture and Cognition of Reporting Guideline. Lanzhou University, 2012. [Article in Chinese]

[20] Dawes JG. Do data characteristics change according to the number of scale points used? An experiment using 5 point, 7 point and 10 point scales. Int J Mark Res. 2008;51(1).

[21] Acupuncture. Available at: https://en.wikipedia.org/wiki/Acupuncture. Accessed 6 Jan 2016.

[22] Birch S, Felt R (1999). Understanding acupuncture.

[23] Western medical acupuncture: a definition. Available at: http://your-gp.com/western-acupuncture-vs-traditional-acupuncture/. Accessed 6 Jan 2016.

[24] Jiang M, Lu C, Zhang C, Yang J, Tan Y, Lu A (2012). Syndrome differentiation in modern research of traditional Chinese medicine. J Ethnopharmacol.

[25] http://www.acupuncturemoxibustion.com/acupuncture/nine-classical-needles/. Accessed 6 Jan 2016.

[26] Zhang TS, Jin CN, Guan F (2009). The history and development of new nine-needle. Chin Acupunct Moxibustion.

[27] Ernst E, Lee MS, Choi TY (2011). Acupuncture: does it alleviate pain and are there serious risks? A review of reviews. PAIN®.

[28] Xu S, Wang L, Cooper E, Zhang M, Manheimer E, Berman B, et al. Adverse events of acupuncture: a systematic review of case reports. Evid Based Complement Alternat Med. 2013;2013. 10.1155/2013/581203 PMC3616356 23573135

[29] Sackett DL, Rosenberg WM, Gray JA, Haynes RB, Richardson WS (1996). Evidence based medicine: what it is and what it isn't. BMJ.

[30] Nulty DD (2008). The adequacy of response rates to online and paper surveys: what can be done?. Assess Eval High Educ.

[31] Asch DA, Jedrziewski MK, Christakis NA (1997). Response rates to mail surveys published in medical journals. J Clin Epidemiol.

[32] Lin ML. Comparison of the Present Situations of Acupuncture and Moxibustion among China Mainland and Taiwan Region. Nanjing University of Chinese Medicine. 2007. [Article in Chinese]

[33] Li ZC (2009). Analysis on Resource Allocation and Utilization in Township Hospitals Under Health Reform in China. Chinese Health Econ.

