# Supplementary material for: A survey of evidence users about the information need of acupuncture clinical evidence
Source: BMC Complement Altern Med. 2016 Nov 10;16:455. doi: 10.1186/s12906-016-1434-0 (PMC5103408; doi:10.1186/s12906-016-1434-0)
Supplement: Additional file 1: — The copy of our questionnaire. (DOC 44 kb) [file 12906_2016_1434_MOESM1_ESM.doc]

**A Survey of Evidence Users about the Information Need of Acupuncture Evidence**

*Respectful respondents：*

Thanks for spending your precious time to participate in this survey! This questionnaire aims at collect the opinions on the “information need” with acupuncture evidence, especially systematic review and meta analysis, from the perspective of clinical practitioners, researchers, and stakeholders in acupuncture area. Please mark **√** or fill the blank to give your answer [choice questions are single selection if no special instruction provided]. Your participation is the greatest support to us, thank you again!

EBM Center of Lanzhou University. Acupuncture research group

2013.12

**Basic Information**: Education ________; Gender: _______ Occupation: _______; Professional title ________; Age_____; Specialty _________

**1. How long have you been working with acupuncture?**

A. <5 years; B. 5-10 years; C. 10-20 years; D. >20 years

**2. What’s your main work related to acupuncture? (multi-selection)**

A. clinical treatment; B. rehabilitation care; *conducting*: C. clinical study D. basic research E. review F. systematic review/ meta analysis G. clinical guideline; H. peer review; I. clinical guideline; J. others _________

**3. How many papers on acupuncture you read per month?**

A. >10 B. 5-10 C. 1-5 D. 0

**4. Where do you obtain the important information in acupuncture? (multi-selection)**

A. electronic databases B. professional journals C. academic conference D. website E. ancient literature F. others______

**5. Which type(s) of literature you usually read? (multi-selection)**

A. RCTs B. observational study C. basic research F. review E. systematic review/meta analysis C. clinical guideline E ancient literature E. others______

**6. Are you satisfied with the completeness of the information reported in acupuncture systematic review/meta analysis?**

A. very satisfied B. basically satisfied C. occasionally satisfied D. not satisfied

**7. Which items should be reported in acupuncture systematic review/meta analysis [Note: there are** 10 different importance levels [eg □1, □2, □3, □4, □5, □6, □7, □8, □9, □10], in which **10 stand for the most important and 1 stand for the least important**, and the importance of the items increases by number. Please mark your level with **√** and give your reasons. Thanks!]

**7.1** Provide the theoretical basis of acupuncture use in target disease in background/introduction.

□1, □2, □3, □4, □5, □6, □7, □8, □9, □10 reason __________________________

**7.2** Provide the style of acupuncture treatment (e.g. traditional Chinese acupuncture, South Korea acupuncture) in background/introduction.

□1, □2, □3, □4, □5, □6, □7, □8, □9, □10 reason __________________________

**7.3** Provide the diagnostic criteria in methods (TCM syndrome and/or diagnostic criteria of diseases in western medicine).

□1, □2, □3, □4, □5, □6, □7, □8, □9, □10 reason __________________________

**7.4** Provide types of acupuncture interventions in methods (e.g. type of acupunctures like percussopunctator and needles, and any other intervention like sham acupuncture)

□1, □2, □3, □4, □5, □6, □7, □8, □9, □10 reason __________________________

**7.5** Provide details of acupuncture interventions in methods (e.g. number of needles, names of acupoint, depth of puncture, relevant body response, needling manipulation, time for needle retention, types of needles)

□1, □2, □3, □4, □5, □6, □7, □8, □9, □10 reason __________________________

**7.6** Provide indicators of effect judgement in methods (e.g. TCM symptomatic relief, Visual Analogue Scale (VAS)).

□1, □2, □3, □4, □5, □6, □7, □8, □9, □10 reason __________________________

**7.7** Provide the qualification (e.g. career and other experience) of acupuncture clinicians in methods.

□1, □2, □3, □4, □5, □6, □7, □8, □9, □10 reason __________________________

**7.8** Provide the follow-up time along with rationality in results.

□1, □2, □3, □4, □5, □6, □7, □8, □9, □10 reason __________________________

**7.9** What additional information do you think must be reported?

Item and reason __________________________ □1, □2, □3, □4, □5, □6, □7, □8, □9, □10

Item and reason __________________________ □1, □2, □3, □4, □5, □6, □7, □8, □9, □10
